# Design of Highly Sensitive C_2_H_5_OH Sensors Using Self-Assembled ZnO Nanostructures

**DOI:** 10.3390/s111009685

**Published:** 2011-10-12

**Authors:** Kang-Min Kim, Hae-Ryong Kim, Kwon-Il Choi, Hyo-Joong Kim, Jong-Heun Lee

**Affiliations:** Department of Materials Science and Engineering, Korea University, Seoul 136–713, Korea; E-Mails: mackjan@korea.ac.kr (K.-M.K.); kimryong@gmail.com (H.-R.K.); saaryun@korea.ac.kr (K.C.); yoarin@korea.ac.kr (H.-J.K.)

**Keywords:** ZnO, gas sensor, nanostructures, hierarchical structures, self-assembly

## Abstract

Various ZnO nanostructures such as porous nanorods and two hierarchical structures consisting of porous nanosheets or crystalline nanorods were prepared by the reaction of mixtures of oleic-acid-dissolved ethanol solutions and aqueous dissolved Zn-precursor solutions in the presence of NaOH. All three ZnO nanostructures showed sensitive and selective detection of C_2_H_5_OH. In particular, ultra-high responses (*R_a_/R_g_* = ∼1,200, *R_a_*: resistance in air, *R_g_*: resistance in gas) to 100 ppm C_2_H_5_OH was attained using porous nanorods and hierarchical structures assembled from porous nanosheets, which is one of the highest values reported in the literature. The gas response and linearity of gas sensors were discussed in relation to the size, surface area, and porosity of the nanostructures.

## 1. Introduction

The chemo-resistive detection of reducing gases in n-type oxide semiconductors is determined by various factors such as the degree of electron depletion in nanostructures, the electron transfer across the necks or grain boundaries, the effective diffusion of analyte gases onto the sensor surface, the amount of oxygen adsorption on the surface, and the surface reaction between analyte gases and negatively charged adsorbed oxygen [1–8]. Most of the key factors in gas sensing reactions, except the surface reactions, can be effectively manipulated by proper control of the size, morphology, crystallinity, and agglomerated configuration of the nanostructures used. To achieve high gas responses, nanostructures with small size, high surface area, good crystallinity, macro-, meso- and nano-porosity, and less-agglomerated configurations are advantageous [9–13].

ZnO is one of the most representative chemo-resistive n-type oxide semiconductors. Many synthetic routes to prepare ZnO nanostructures have been suggested, which include hydrothermal reactions [14–16], ambient-atmosphere solution reactions at mild temperature (50–100 °C) [17–19] or room temperature [20,21], thermal evaporation [22], and microwave methods [23]. Among these, solution-based self-assembly reactions under ambient atmosphere provide facile and cost-effective methods to prepare various ZnO nanostructures with high surface areas. When the low-dimensional nano-building blocks such as 1-dimensional (1D) nanorods and 2D nanosheets are self-assembled into the higher dimensional hierarchical structures, well-defined porous architectures can be achieved without sacrifice of high surface area. Thus the gas response, as well as the response kinetics, can be enhanced significantly by the rapid and effective diffusion of analyte gases to the entire sensing surface [24–26].

The present authors have previously prepared ZnO hierarchical nanostructures assembled from dense nanosheets by the forced stirring of immiscible mixtures of oleic-acid-dissolved *n*-hexane solutions and aqueous solutions of dissolved Zn-precursors and subsequent addition of NaOH [27]. In this contribution, we further controlled the morphology and porosity of ZnO nanostructures by the reaction of mixtures of oleic-acid-dissolved ethanol solutions and the aqueous solutions of dissolved Zn-precursors in the presence of NaOH. Various nanostructures such as porous nanorods and hierarchical nanostructures consisting of porous nanosheets or crystalline nanorods could be synthesized by employing ethanol in the oleic-acid-dissolving solvent mixture instead of *n*-hexane, by controlling the addition of NaOH, or by mild heating of the solutions. Our main focus was directed at studying the effect of size, crystallinity, surface area, morphology, and nano-porosity of the various nanostructures on the gas response and linearity of the resulting gas sensors.

## 2. Experimental Section

The morphologies of Zn-precursors were controlled by changing the reaction steps and reaction temperatures. Three Zn-precursors with different morphologies were prepared: hierarchical structures assembled from nanosheets (referred as “H-NS” precursor); thin nanorods (“NR” precursor); hierarchical structures assembled from thick nanorods (“H-NR” precursor). These were converted into the corresponding ZnO nanostructure-like morphologies by heat treatment at 500 °C for 1 h, and will be referred to hereafter as the “H-NS”, “NR” and “H-NR” nanostructures, respectively.

### 2.1. Preparation of H-NS Precursors

Zn(NO_3_)_2_·6H_2_O (2.38 g, >99%, Kanto Chemical, Japan) was dissolved in deionized water (160 mL) after which ethanol (C_2_H_5_OH, 40 mL, 99%, Sigma Aldrich, USA) and oleic acid (C_18_H_34_O_2_, 1.28 g, >99%, Sigma Aldrich, USA) were added to the solution in sequence with stirring. While ethanol and oleic acid are miscible with each other, oleic acid is insoluble in aqueous solution. Thus, water-insoluble and ethanol-soluble surfactant (oleic acid) was provided continuously and gradually by the forced stirring of the emulsion mixture of the oleic-acid-dissolved ethanol solution and the dissolved Zn-precursor aqueous solution. After 50% NaOH aqueous solution (6.4 g, Samchun Chemical Co., Korea) was instantaneously poured into the mixture, the resulting emulsion was stirred for 1 h at room temperature.

### 2.2. Preparation of NR Precursors

50% NaOH aqueous solution (6.4 g) was dissolved in deionized water (160 mL). Then Zn(NO_3_)_2_·6H_2_O (2.38 g) dissolved in ethanol (40 mL) and oleic acid (1.28 g) were instantaneously poured into the solution in sequence. The solution was reacted for 1 h with continuous stirring.

### 2.3. Preparation of H-NR Precursors

Zn(NO_3_)_2_·6H_2_O (2.38 g) was dissolved in deionized water (200 mL). After the instantaneous addition of 50% NaOH solution (9.6 g), the solution was heated at 90 °C for 1 h. The resultant products were collected by centrifugation, washed several times with deionized water and ethanol, and dried at room temperature.

### 2.4. Characterization

The phase and crystallinity of the powders were analyzed by X-ray diffraction (XRD, Rigaku D/MAX-2500 V/PC). The morphology of the powders was investigated using field-emission scanning electron microscopy (FE-SEM, S-4800, Hitachi Co. Ltd., Japan). High resolution transmission electron microscopy (HR-TEM) (JEM-2100F) was used to examine the microstructure of the individual powders. To investigate the thermal decomposition of the precursors, Differential scanning calorimetry/thermogravimetric analyses (DSC/TGA) (SDT Q600, Ta instrument, Inc) were carried out under air in the temperature range from room temperature to 700 °C. The surface areas were measured by using the Brunauer–Emmett–Teller (BET) method (Tristar 3000, Micromeritics Co. Ltd.).

### 2.5. Gas Sensing Characteristics

The as-prepared precursors were prepared into a paste form and applied to an alumina substrate (size: 1.5 mm × 1.5 mm, thickness: 0.25 mm) having two Au electrodes (electrode width: 1 mm, electrode spacing: 0.2 mm). The sensor element was heated to 500 °C at 25 °C/min and then treated at this temperature for 1 h for conversion into pure ZnO nanostructures and to decompose the organic content of the paste. The sensor was placed in a quartz tube and the temperature of the furnace was stabilized at 400 °C. A flow-through technique with a constant flow rate of 500 cm^3^/min was used and 4-way valve was employed to switch the gas atmospheres. The gas responses (S = *R_a_/R_g_*, *R_a_*: resistance in dry air, *R_g_*: resistance in gas) to 100 ppm C_2_H_5_OH, CO, H_2_, and C_3_H_8_ were measured at 400 °C. The gas concentration was controlled by changing the mixing ratio of the parent gases (100 ppm C_2_H_5_OH, 100 ppm CO, 100 ppm H_2_, and 100 ppm C_3_H_8_, all in dry air balance) and dry synthetic air. The dc 2-probe resistance of the sensor was measured using an electrometer interfaced with a computer.

## 3. Results and Discussion

**Figure 1.**
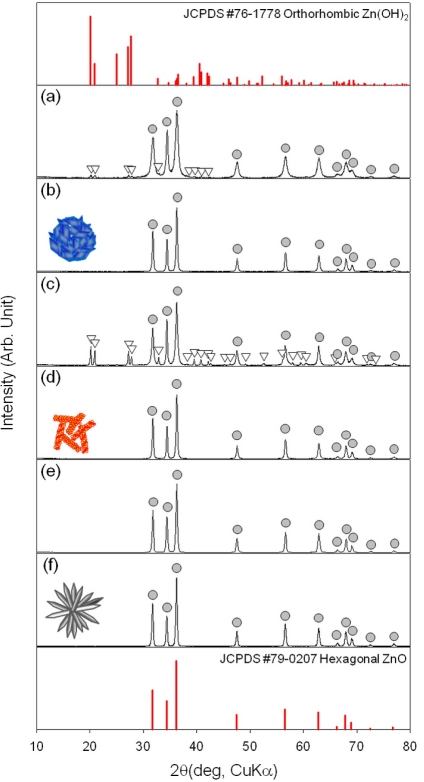
X-ray diffraction patterns of (**a**) H-NS precursors; (**b**) H-NS nanostructures; (**c**) NR precursors; (**d**) NR nanostructures; (**e**) H-NR precursors; and (**f**) H-NR nanostructures. H-NS, NR, and H-NR ZnO nanostructures were prepared by heat treatment of H-NS, NR, and H-HR precursors at 500 °C for 1 h, respectively.

The phase and composition of as-prepared precursors and ZnO nanostructures after heat treatment at 500 °C for 1 h in air were examined by X-ray diffraction (XRD) (Figure 1). The H-NS and NR precursors were identified as the mixture between hexagonal ZnO (JCPDS #79-0207) and orthorhombic Zn(OH)_2_ (JCPDS #76-1778)[Figure 1(a,c)]. The Zn(OH)_2_ phase content was higher in NR precursors. In contrast, the H-NR precursors were identified as crystalline ZnO phase without Zn(OH)_2_ [Figure 1(e)]. All the three precursors were converted into pure ZnO by heat treatment at 500 °C for 1 h [Figure 1(b,d,f)].

**Figure 2.**
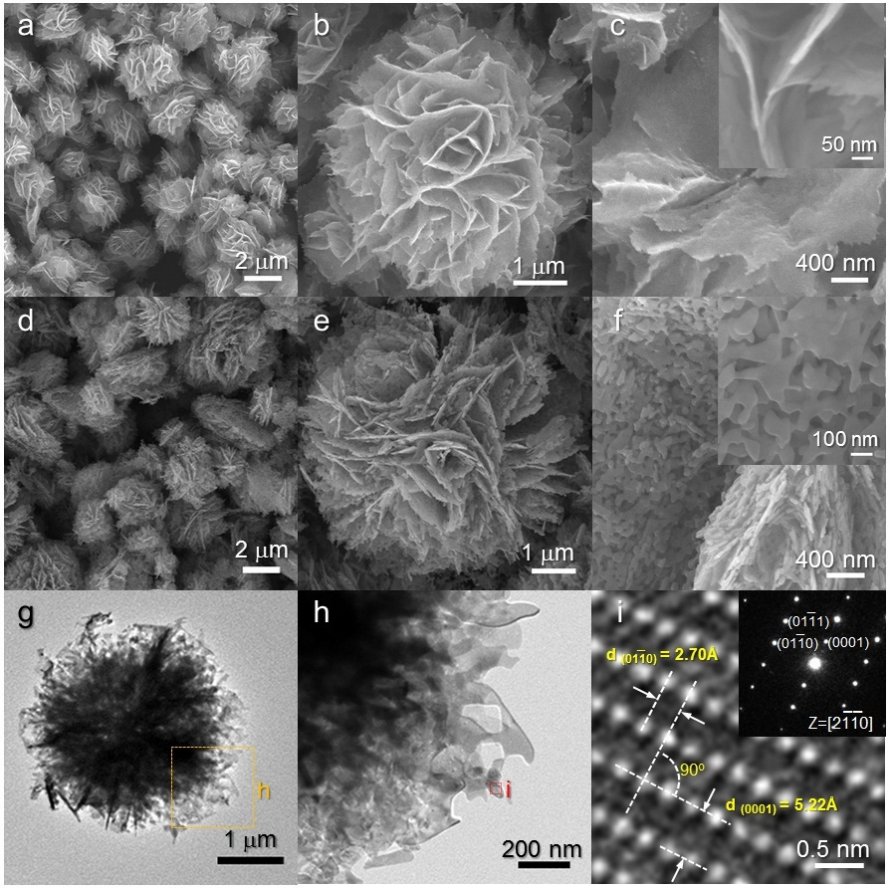
(**a–c**) SEM images of as-prepared H-NS precursors; (**d–f**) SEM images of heat-treated H-NS ZnO nanostructures; (**g–i**) TEM images of heat-treated H-NS ZnO nanostructures.

As-prepared H-NS precursors were hierarchical structures assembled from nanosheets [Figure 2(a,b)]. The sizes of assembled hierarchical structures ranged from 3 to 5 μm. Closer inspection revealed that the 2-dimensional nano-building blocks (nanosheets) are extremely thin (5–10 nm) [Figure 2(c)]. The overall hierarchical morphology was maintained after heat treatment at 500 °C for 1 h [Figure 2(d,e)]. However, the relatively smooth surfaces of the as-prepared precursors [Figure 2(c)] were changed into nano-porous ones upon heat treatment [Figure 2(f)]. The nano-porous and sheet-like structures were further confirmed by TEM analysis [Figure 2(g,h)]. Well-developed crystalline structures were found in the local area [Figure 2(i)]. Two lattice planes with interplanar distances of 2.70 and 5.22 Å and an angle of 90 ° were observed in the lattice fringe, which corresponded to the (10–10) and (0001) planes of the ZnO (hexagonal) crystal structures, respectively.

**Figure 3.**
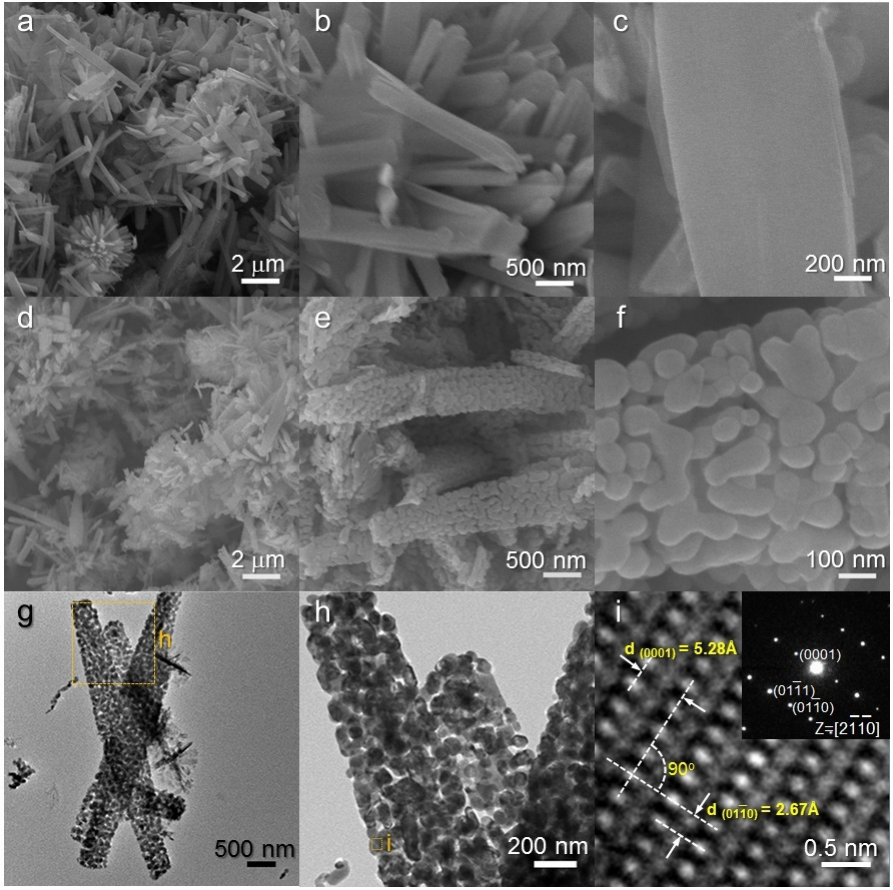
(**a–c**) SEM images of as-prepared NR precursors; (**d–f**) SEM images of heat-treated NR ZnO nanostructures; (**g–i**) TEM images of heat-treated NR ZnO nanostructures.

The NR precursors consist of 1-dimensional nanorods [Figure 3(a–c)]. These nanorods were typically 2–3 μm long and 300–700 nm thick. Note that the surfaces of NR precursors are relatively clean and smooth [Figure 3(c)]. The rod morphology remained similar after heat treatment at 500 °C for 1 h [Figure 3(d–f)]. However, nano-porous structures developed on the surface of nanorods by heat treatment [Figure 3(f)]. The low and high magnification TEM images [Figure 3(g,h)] confirm again that nanorods are nano-porous and consist of small primary particles (size: 30 to 200 nm). Lattice image of local area showed that each primary particle is highly crystalline ZnO [Figure 3(i)].

**Figure 4.**
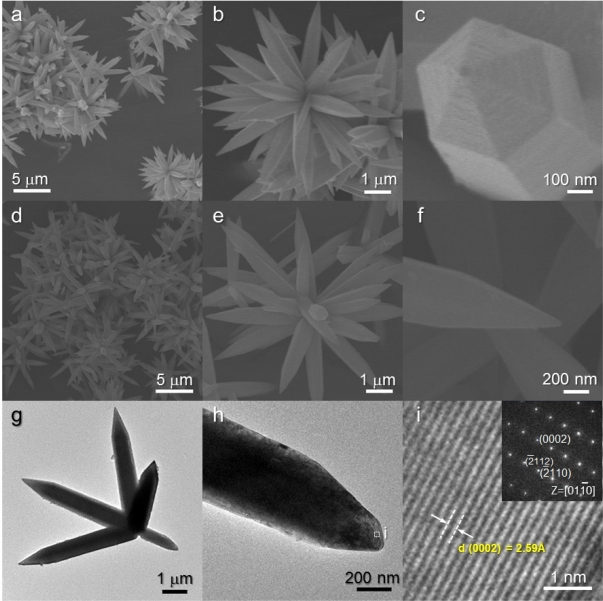
(**a–c**) SEM images of as-prepared H-NR precursors; (**d–f**) SEM images of heat-treated H-NR ZnO nanostructures; (**g–i**) TEM images of heat-treated H-NR ZnO nanostructures.

The H-NR precursors were hierarchical structures assembled from nanorods [Figure 4(a–c)]. The relatively thick nanorods (typical thickness: ∼700 nm) with sharp edges were hierarchically assembled into an urchin-like morphology. A closer look revealed the hexagonal structures of the nanorods [Figure 4(c)]. This morphology was also found in other ZnO hierarchical structures prepared from different physico-chemical routes [28,29] and indicates the growth of highly crystalline ZnO nanorods along the [0001] direction. The hierarchical structures were maintained after heat treatment at 500 °C [Figures 4(d–f)]. The nanorods with sharp edges were identified as single crystalline ZnO by TEM analysis [Figures 4(g–i)].

It should be noted that the surfaces of heat-treated H-NR nanostructures remained smooth and dense after heat treatment [Figure 4(d–f)] while those of heat-treated H-NS and NR nanostructures were changed into nano-porous ones by the heat treatment [Figures 2(f) and 3(f)]. Considering the phases of H-NS [ZnO + Zn(OH)_2_] and NR [ZnO + Zn(OH)_2_], and H-NR precursors (pure ZnO) [Figure 1(a,c,e)], the development of nano-porous structures in the H-NS and NR nanostructures [Figures 2(f) and 3(f)] is thought to be related to the dehydration of the Zn(OH)_2_ phase during heat treatment.

**Figure 5.**
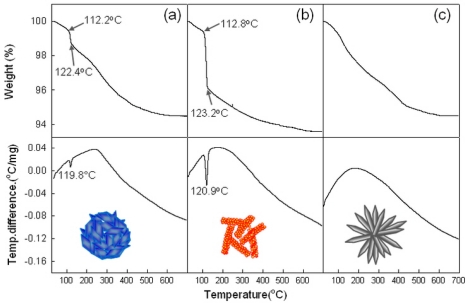
Differential scanning calorimetry (DSC) and thermogravimetric analysis (TGA)curves of (**a**) H-NS precursors; (**b**) NR precursors; and (**c**) H-NR precursors.

To confirm above idea, the thermal evolutions of precursors were analyzed using DSC and TGA (Figure 5). The sharp endothermic peaks at ∼120 °C [Figure 5(a,b)] and the abrupt weight loss between 112–123 °C in H-NS and NR precursors can be attributed to the dehydration of Zn(OH)_2_ phase. This can be supported by the higher weight loss in NR precursors containing the more Zn(OH)_2_ phase [Figure 5(b)]. Indeed, neither the endothermic peak nor the abrupt weight loss near 112–123 °C was found in H-NR precursors [Figure 5(c)] without Zn(OH)_2_ phase.

**Figure 6.**
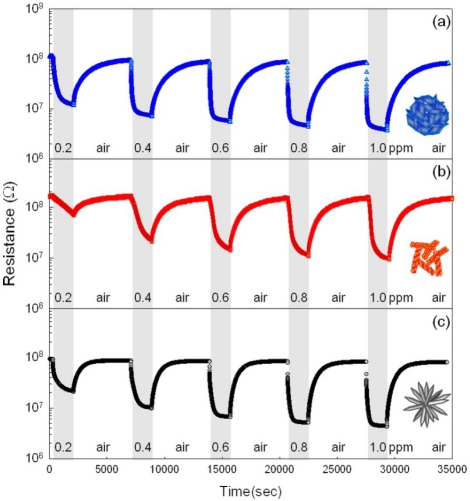
C_2_H_5_OH sensing transients of (**a**) H-NS sensor; (**b**) NR sensor; and (**c**) H-NR sensor at 340 °C. (C_2_H_5_OH concentration: 0.2–1 ppm).

The gas responses to 100 ppm C_2_H_5_OH, C_3_H_8_, CH_4_, CO and H_2_ were measured at 250–400 °C. The response to C_2_H_5_OH was higher than those to other gases and the highest gas response was attained at 340 °C (data not shown). Thus, the dynamic sensing transients to 0.2–1 ppm C_2_H_5_OH were measured at 340 °C (Figure 6). All the H-NS, NR and H-NR sensors showed very high responses, even to sub-ppm-level C_2_H_5_OH, and sensor resistances recovered to the air-level value reproducibly. The times to reach 90% variation in sensor resistance upon exposure to gas was defined as 90% response time (*τ_res_*). The *τ_res_* value of NR sensor (1,214 s) upon exposure to 1 ppm C_2_H_5_OH and air were higher than those of the H-NS (866 s) and H-NR (500 s) sensors. The NR precursors contained the largest amount of Zn(OH)_2_, while no Zn(OH)_2_ phase was found in H-NR precursors. Thus, the slowest response in NR sensor may be explained either by the change of nano-porosity or by the variation of trace amount of residual (OH)^−^ radicals after heat-treatment. Longer times were required for response as the C_2_H_5_OH concentration decreased down to 0.2 ppm. Although the highest gas responses were attained at 340 °C, relatively sluggish response and recovery kinetics can limit the application of this sensor.

**Figure 7.**
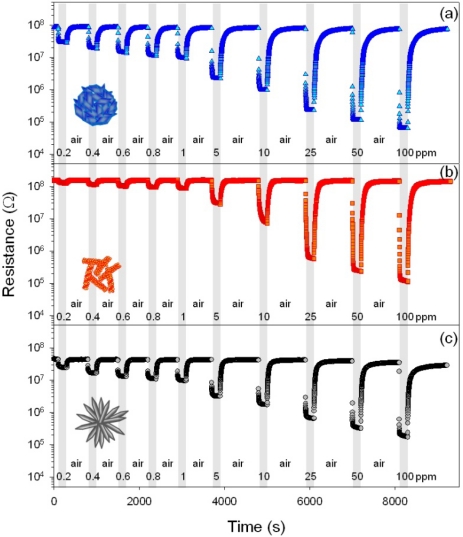
C_2_H_5_OH sensing transients of (**a**) H-NS sensor; (**b**) NR sensor; and (**c**) H-NR sensor at 400 °C. (C_2_H_5_OH concentration: 0.2–100 ppm).

In order to enhance the response and recovery rates, the sensor temperature was increased to 400 °C. Figure 7 shows the sensing transients to 0.2–100 ppm C_2_H_5_OH at 400 °C (Figure 7). In all the three sensors, the gas responses to C_2_H_5_OH decreased with increasing sensor temperature. Nevertheless, the responses to 0.2–1 ppm C_2_H_5_OH of H-NS (*R_a_/R_g_* =3.1–8.9), NR (*R_a_/R_g_* = 1.2–1.8), and H-NR sensors (*R_a_/R_g_* = 1.9–2.5) were still very high and enough to detect sub-ppm-levels of C_2_H_5_OH. The *τ_res_* value of the NR sensor upon exposure to 1 ppm C_2_H_5_OH and air were markedly decreased to 1.8 s and the *τ_res_* values of H-NS and H-NR sensors were also decreased significantly to 8.9 and 4.5 s, respectively. Taking into account both of gas response and gas responding speed, the operation of the sensor at 400 °C is more advantageous.

**Figure 8.**
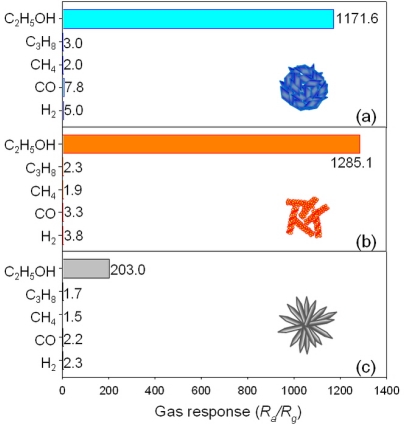
Gas responses to 100 ppm C_2_H_5_OH, C_3_H_8_, CH_4_, CO and H_2_ of (**a**) H-NS sensor; (**b**) NR sensor; and (**c**) H-NR sensor at 400 °C.

The gas responses to 100 ppm C_2_H_5_OH, C_3_H_8_, CH_4_, CO and H_2_ at 400 °C were compared (Figure 8). The responses to 100 ppm C_2_H_5_OH of H-NS and NR sensors were 1,171.6 and 1,285.1, respectively, which were significantly higher than those to C_3_H_8_, CH_4_, CO and H_2_ [Figure 8(a,b)]. Although the response to 100 ppm C_2_H_5_OH of H-NR sensor (203.0) was smaller than those of H-NS and NR sensors, it is still sufficiently higher than the responses to other gases (1.5–2.3) [Figure 8(c)]. Accordingly, all the sensors in the present study can be used for the selective detection of C_2_H_5_OH with minimum cross-sensitivities to C_3_H_8_, CH_4_, CO and H_2_. High selectivity to C_2_H_5_OH may be attributed to the higher chemical interaction between C_2_H_5_OH and ZnO surface, the more active electrochemical interaction between C_2_H_5_OH and O^−^ on the surface of ZnO.

**Figure 9.**
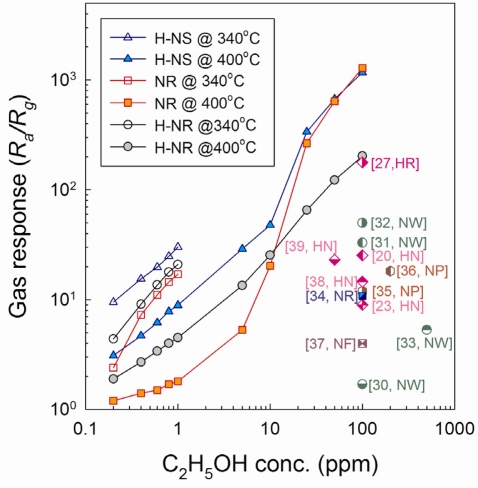
Gas responses to 0.2–100 ppm C_2_H_5_OH of H-NS, NR, and H-NR sensors at 340 and 400 °C in the present study and other pure ZnO nanostructured sensors in the literature [19,23,30–39]. (HN: hierarchical nanostructures, NW: nanowires, NP: nanoparticles, NR: Nanorods, NF: nanofibers).

The responses to C_2_H_5_OH of the sensors at 340 and 400°C and those of various ZnO nanostructures in the literature [19,23,30–39] were plotted in Figure 9. At the sensor temperature of 340 °C, the H-NS sensor showed the highest responses to 0.2–1 ppm C_2_H_5_OH, followed by NR sensor and H-NR sensor. The same order was also found in the gas responses to sub-ppm-level C_2_H_5_OH at 400 °C although the absolute response values were decreased. However, the order of gas responses was changed as increasing C_2_H_5_OH concentration to 25–100 ppm. In the log-log plot, the gas responses of H-NS and NR sensors abruptly increase near 5–25 ppm C_2_H_5_OH, while those of H-NR sensors increase linearly with the entire range of concentration. As a result, the responses to 25–100 ppm C_2_H_5_OH of NR sensors become larger than those of H-NR sensors.

**Figure 10.**
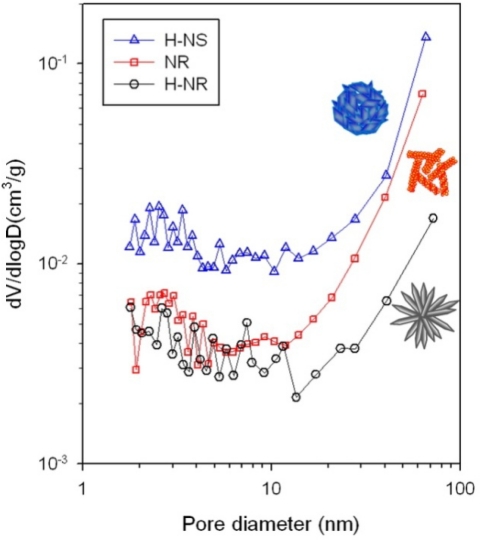
Pore size distributions of heat-treated H-NS, NR, and H-NR ZnO nanostructures determined from the nitrogen adsorption-desorption isotherm.

The pore size and volume distribution and surface area were analyzed by nitrogen adsorption-desorption isotherm measurements (Figure 10). The surface areas of H-NS, NR and H-NR nanostructures after heat treatment at 500 °C for 1 h were 17.2, 7.7 and 4.5 m^2^/g, respectively. The pore volumes of H-NS nanostructures over the entire pore sizes were substantially higher than those of NR and H-NR nanostructures, which agree well with the highest gas response of H-NS sensor. The pore volumes of NR nanostructures in the size range of 2–10 nm are slightly larger than those of H-NR nanostructures, whereas the pore volumes of NR nanostructures in the size range of 10–100 nm are significantly higher than those of H-NR nanostructures. The order of gas response values to 25–100 ppm C_2_H_5_OH can be explained by the different surface areas available for gas sensing. However, at glance, it is difficult to explain why the H-NR sensors with lower surface area (4.5 m^2^/g) show the higher responses to sub-ppm-level C_2_H_5_OH than the NR sensors with a higher surface area (7.7 m^2^/g). Although further study is needed, a plausible explanation can be given as follows: at low concentration range, the gas sensing reaction may occur more effectively on the surface of highly crystalline H-NR sensors rather than on the surface of nano-porous and polycrystalline NR sensor. The amount of oxygen adsorption can vary according to the crystallographic planes of oxide nanocrystals, which is supported by the literature [40] which states that the gas responses depend closely on the preferred orientation of ZnO nanocrystals. Thus, the high response of H-NR sensors despite its low surface area might be attributed to the enhanced gas sensing behaviors at the specific crystallographic plane.

It should be noted that non-linear gas response behaviors are only found at the H-NS and NR sensors with nano-porous structures developed from the heat-treatment of hydroxide precursors. At low concentration, most of the analyte gas will be consumed by the reaction with negatively charged oxygen on the outermost surfaces. However, as analyte concentration increases, excess analyte gas can diffuse further into the inner part of nanopores, which provides an additional contribution to the gas sensing reaction. Thus, the non-linear sensing behaviors at high C_2_H_5_OH concentration can be explained by the additional gas sensing reaction within the nanopores.

At the sensing temperature of 400°C, the low detection limit of C_2_H_5_OH of H-NS sensor was estimated to be <0.057 ppm from the extrapolation of the linear part at the low concentration range (0.2 to 10 ppm C_2_H_5_OH), when the criterion for gas detection was set to *R_a_/R_g_* > 1.2. This demonstrates that the present sensor can be used to detect the several-tens-ppb-levels of C_2_H_5_OH. Moreover, the deviation of gas responses from the linear regime at the high C_2_H_5_OH concentration range leads to ultra-high gas responses. The gas responses of H-NS and NR sensors to 100 ppm C_2_H_5_OH are ∼1,200, which are among the highest values reported in the literature for ZnO nanowires [30–33], nanorods [34], nanoparticles [35,36], nanofibers [37], and hierarchical nanostructures [19,23,27,38,39] (Figure 9). The linear sensing behaviors over the concentration range between 0.2 to 100 ppm could be obtained using H-NR sensor. These clearly show that the high gas response and linearity can be effectively designed by controlling the size, morphology, and macro- and nano-porosity of the nanostructures.

## 4. Conclusions

Three different morphologies of ZnO nanostructures for gas sensor applications were prepared by controlling the solvent to dissolved surfactant, the NaOH addition procedure, and the solution temperature during an oleic-acid-based self-assembly reaction. In the log-log plot of gas responses and analyte (C_2_H_5_OH) concentration, the hierarchical structures assembled from dense and crystalline nanosheets showed linear sensing behaviors. In contrast, the gas responses of porous nanorods and hierarchical structures assembled from porous nanosheets showed a deviation from the linear line above 5–10 ppm C_2_H_5_OH and lead to ultra-high responses (*R_a_/R_g_* = ∼1,200, *R_a_*: resistance in air, *R_g_*: resistance in gas) to 100 ppm C_2_H_5_OH. This non-linearity of gas responses at high C_2_H_5_OH concentration was attributed to the additional gas sensing reaction occurring within the nanopores by the diffusion of excess analyte gas into the nanopores. The results show that not only the linear sensing but also the ultra-high gas response can be effectively designed by the control of size, morphology, and porosity of nanostructures.
